# Constitutively Active CCR5 Chemokine Receptors Differ in Mediating HIV Envelope-dependent Fusion

**DOI:** 10.1371/journal.pone.0054532

**Published:** 2013-01-23

**Authors:** Alex de Voux, Mei-Chi Chan, Asongna T. Folefoc, Michael T. Madziva, Colleen A. Flanagan

**Affiliations:** 1 Medical Research Council Receptor Biology Research Unit, Division of Medical Biochemistry, Faculty of Health Sciences, University of Cape Town, Cape Town, South Africa; 2 Medical Research Council Receptor Biology Research Unit, School of Physiology, University of the Witwatersrand, Johannesburg, South Africa; Medical School of Hannover, United States of America

## Abstract

The CCR5 chemokine receptor is a rhodopsin-like G protein-coupled receptor that mediates the effects of pro-inflammatory β-chemokines. CCR5 is also the major co-receptor for entry of human immunodeficiency virus (HIV) into human cells. G protein-coupled receptors exist in ensembles of active and inactive conformations. Active receptor conformations can be stabilized by mutations. Although binding of the HIV envelope protein to CCR5 stimulates cellular signaling, the CCR5 conformation that induces fusion of the viral membrane with cellular membranes is not known. We mutated conserved amino acids to generate constitutively active CCR5 receptors, which are stabilized in active conformations, and tested the ability of constitutively active CCR5 receptors to mediate HIV envelope-directed membrane fusion. Mutation of the Asp^3.49(125)^ and Arg^6.32(225)^ residues of CCR5 did not cause constitutive activity, but Lys or Pro substitutions for Thr^2.56(82)^, in the TxP motif, caused high basal inositol phosphate signaling. Signaling did not increase in response to MIP-1β, suggesting that the Thr^2.56(82)^ mutants were fully stabilized in active conformations. The Thr^2.56(82)^Lys mutation severely decreased cell surface CCR5 expression. Combining the Thr^2.56(82)^Lys mutation with an Arg^6.32(225)^Gln mutation partially reversed the decrease in expression. Mutants with Thr^2.56(82)^Lys substitutions were poor mediators of HIV envelope-directed membrane fusion, but mutants with the Thr^2.65(82)^Pro substitution exhibited full co-receptor function. Our results suggest that the Thr^2.65(82)^Lys and Thr^2.65(82)^Pro mutations stabilize distinct constitutively active CCR5 conformations. Lys in position 2.65(82) stabilizes activated receptor conformations that appear to be constitutively internalized and do not induce envelope-dependent membrane fusion, whereas Pro stabilizes activated conformations that are not constitutively internalized and fully mediate envelope-directed membrane fusion.

## Introduction

The CCR5 chemokine receptor is a G protein-coupled receptor (GPCR) that mediates leukocyte chemotaxis and recruitment to sites of inflammation in response to pro-inflammatory β-chemokines, including macrophage inflammatory protein 1β (MIP-1β, CCL4) [1], [2]. CCR5 is also the major co-receptor for human immunodeficiency virus (HIV) infection. Sequential binding of the surface gp120 subunit of the HIV envelope glycoprotein (Env) to cellular CD4 and CCR5 induces a “fusogenic” Env conformation that penetrates the cell membrane and fuses the viral and cellular membranes. The CCR5 chemokine receptor is an attractive target for treatment and prevention of HIV infection and the first CCR5-blocking drug, maraviroc, was approved in 2007.

GPCR proteins exist in ensembles of inactive conformations, which are stabilized by inverse agonists and do not support intracellular signaling, and active receptor conformations, which are stabilized by agonists and activate corresponding ensembles of cellular signaling pathways. Ligands may selectively stabilize ensembles of receptor conformations that activate subsets of cellular signaling pathways [3], [4]. For example, chemokines stabilize CCR5 receptor conformations that activate G protein signaling and conformations that are recognized by G protein-coupled receptor kinases and arrestins, which promote receptor internalization. Some chemokine ligands have distinct efficacies for stimulating intracellular signaling and internalization of CCR5 [5]. HIV binding to CCR5 must stabilize a receptor conformation that induces the fusion conformation of Env. HIV also stimulates CCR5-dependent cellular signaling [6], [7], [8].

The structures of a small number of GPCR proteins have been determined in inverse agonist-bound inactive conformations [9], [10], [11], [12] and in complexes with agonist and a G protein or G protein mimetic, which stabilize active receptor conformations [13], [14], [15]. The crystal structures support hypotheses that amino acids that are highly conserved among GPCRs form distinct intramolecular interactions in active and inactive receptor conformations and act as activation “switches” [4], [16], [17], [18].

Supporting the switch hypothesis, mutation of the Asp^3.49^ and Arg^3.50^ residues of the conserved DRY (Asp-Arg-Tyr) motif, in transmembrane segment (TMS) 3, stabilizes mutant receptors in activated conformations, which stimulate cellular signaling in the absence of agonist [19]. Different mutations of the Thr^2.56(82)^ and Pro^2.58(84)^ residues of the conserved TxP motif, stabilized CCR5 mutants in inactive [20] or constitutively active conformations [21]. A naturally-occurring Arg^6.32(225)^Gln mutation causes partial constitutive activity in CCR5 [22].

The CCR5 conformation(s) that induce the fusogenic changes in Env are not known. Binding of the gp120 subunit of Env to CCR5 stimulates intracellular signaling [6], [7], [8], suggesting that HIV stabilizes activated CCR5 conformations that activate G proteins and other cytosolic signaling proteins. On the other hand, CCR5 receptors with inactivating mutations, which uncouple CCR5 from activation of G protein and other signaling pathways, mediated Env-dependent membrane fusion [23], [24], [25], suggesting that inactive CCR5 conformations mediate HIV entry. Small molecule CCR5-binding anti-HIV drugs are inverse agonists. HIV strains that are resistant to CCR5 “blockers” use drug-bound CCR5 to infect cells [26], [27], [28], [29], suggesting that a drug-stabilized, inactive receptor conformation mediates infection. Thus, inactive CCR5 conformation(s) mediate HIV infection and we hypothesized that activated conformations that stimulate G protein signaling would be poor mediators of Env-directed membrane fusion.

We have investigated the ability of activated conformations of CCR5 to mediate Env-directed membrane fusion by mutating conserved “switch” residues of the human CCR5 chemokine receptor. Mutation of Asp^3.49(125)^ and Arg^6.32(225)^ did not increase constitutive activity. CCR5 mutants with Pro or Lys substituted for Thr^2.56(82)^ showed high basal cellular signaling, which was not increased by stimulation with MIP-1β. The Thr^2.56(82)^Lys mutation decreased cell surface CCR5 protein, whereas the Thr^2.56(82)^Pro mutation did not. Constitutively active CCR5 receptors differed in their ability to mediate Env-directed membrane fusion. Our results suggest that Pro and Lys substitutions in position 2.56(82) stabilize distinct activated CCR5 conformations that differ in their localization at the cell surface and in their ability to induce HIV Env-dependent membrane fusion.

## Materials and Methods

### DNA Constructs, Cell Lines and Proteins

The chimeric G protein construct, Gαqi, which allows receptors that usually activate the Gi/o family of G proteins to stimulate inositol phosphate (IP) signaling [30] was prepared by site-directed mutagenesis, cloned into the pcDNA3.1(+) expression vector (Invitrogen, Carlsbad, CA) and stably expressed in HEK 293 cells (HEK-Gqi) as previously described [22]. The HIV-1C *env* construct pTHr.gp150CT [31] was a gift from Carolyn Williamson (University of Cape Town). The codon-optimized, carboxy-terminally truncated Du151 *env*, Du151 gp150, was subcloned into the pcDNA3.1(+) expression vector (Invitrogen). The HIV-1 *tat* (GenBank Accession number X07861) cloned into pcDNA3.1, HIV-1 *rev* (GenBank Accession No. M34378) cloned into pcDNA3.1/Hygro (Invitrogen) and the pHIV-1LTR-Luc reporter construct [32] were gifts from Steven Jenkinson, GlaxoSmithKline. The following cell lines were obtained from the AIDS Research and Reference Reagent Program, Division of AIDS, NIAID, NIH: Human osteosarcoma cells stably expressing CD4 (HOS-CD4.pBABE-puro) or CD4 and CCR5 (HOS-CD4-CCR5) from Dr Nathaniel Landau [33]. The pHIV-1LTR-Luc construct was stably transfected into both of these cell lines. Recombinant human chemokine MIP-1β (CCL4) was purchased from Peprotec (Rocky Hill, NJ).

### Generation of Mutant CCR5 Receptor Constructs

Mutant CCR5 receptor constructs were generated by PCR using Deep Vent high fidelity DNA polymerase (New England Biolabs, Ipswich, MA) and the wild type human CCR5 chemokine receptor cDNA, cloned into the pcDNA3.1(+) expression vector (Invitrogen, Carlsbad, CA), as template. The Ballesteros and Weinstein amino acid numbering system [34] is used to facilitate comparison of CCR5 with other rhodopsin-like GPCRs. The generic residue number consists of the TMS, 1 to 7, in which the residue is located, followed by the position relative to the most conserved residue of the TMS, which is designated number 50. The generic number is followed by the number of the residue in the sequence of the CCR5 receptor. For example, the Asp^125^ residue in the conserved DRY motif of the CCR5 receptor is designated Asp^3.49(125)^, because it immediately precedes the most conserved residue in TMS3, Arg^3.50(126)^. Asp^3.49(125)^ was mutated to Ala (Asp^3.49(125)^Ala) and Asn (Asp^3.49(125)^Asn), whereas the Thr^2.56(82)^ residue in TMS2 of CCR5 was mutated to Pro (Thr^2.56(82)^Pro), Lys (Thr^2.56(82)^Lys) and Arg (Thr^2.56(82)^Arg) and Arg^6.32(225)^, in the third intracellular loop, was mutated to Gln, Ala, Asp and Glu. The Arg^6.32(225)^Gln construct was used as the template for the double mutants, Thr^2.56(82)^Lys/Arg^6.32(225)^Gln and Thr^2.56(82)^Pro/Arg^6.32(225)^Gln. Mutant constructs were sequenced and subcloned into the pcDNA3.1(+) and pcDNA3.1/Hygro(+) expression vectors.

### Cell Culture and Transfection

HEK 293 cells (ATCC) were maintained in Dulbecco’s Modified Eagle’s Medium (DMEM, Gibco, Invitrogen, Paisley, Scotland) containing fetal bovine serum (FBS, 10%, Highveld Biologicals, Johannesburg, South Africa) and cultured at 37°C with 10% CO_2_. HEK-Gqi cells were maintained in DMEM supplemented with FBS (10%) and G418 (200 µg/ml). HOS-CD4.pBABE-puro and HOS-CD4-CCR5 cells were maintained in DMEM supplemented with FBS (10%) and puromycin (1 µg/ml), whereas the same cell lines stably transfected with pHIV-1LTR-Luc to generate the cell lines, HOS-CD4-Luc and HOS-CD4-CCR5-Luc, were maintained with FBS, puromycin (1 µg/ml) and G418 (400 µg/ml).

Cells were plated into 10 cm^2^ dishes (3–6×10^6^ cells, Corning, Cambridge, USA) in a final volume of 10 ml DMEM with FBS (10%) 24 h before transfection. DNA constructs (6 µg) were incubated with FuGene HD (30 µl, Roche Diagnostics Corp., Indianapolis, USA) in serum-free DMEM (room temperature, 30 min) and added directly to the 10 ml medium in the 10 cm dishes. Cells were incubated overnight (37°C; 5% CO_2_). For stable transfections, selection antibiotics were added two days later and individual colonies of antibiotic-resistant cells were harvested and propagated. Attempts to stably transfect CCR5 constructs into HOS-CD4-Luc cells were unsuccessful. HOS-CD4-Luc cells transiently transfected with wild type and mutant CCR5 constructs were cultured in the presence of hygromycin B (200 µg/ml) for two days to increase the proportion of receptor-expressing cells and thus compensate for low transfection efficiency.

### IP Production

Basal and MIP-1β-stimulation of IP second messenger production was assessed as previously described [22], [35]. Briefly, HEK-Gqi cells (3×10^6^ per 10 cm dish), transfected with wild type or mutant CCR5 receptor constructs, were distributed into 12-well plates (Corning, 2 plates/10 cm dish), incubated overnight and then incubated with ^3^[H]*myo*-inositol (1 µCi/ml, Amersham Life Sciences, Buckinghamshire, England, 16–18 h). The resulting radio-labeled cells were pre-incubated with buffer I (40 mM NaCl, 4 mM KCl, 20 mM HEPES, 8.3 mM glucose, 1 mM CaCl_2_, 1 mM MgCl_2_, 10 mM LiCl, 0.1% BSA, 0.4% phenol red, 15 min, 37°C) and then incubated in duplicate with buffer I containing various concentrations of MIP-1β (0–10^−7 ^M, 60 min, 37°C), after which the medium was replaced with pre-cooled formic acid (1 ml, 10 mM, 30 min, 4°C). The resulting cell lysates were applied to ion exchange columns (DOWEX-1, Sigma, Bellefonte, USA) and [^3^H]IP was eluted (1 M ammonium formate, 0.1 M formic acid) into vials containing scintillation fluid (16 ml, Quicksafe; Zinsser Analytical, Frankfurt, Germany) and counted. MIP-1β concentrations that stimulated half-maximal IP production (EC_50_ values) were calculated using GraphPad Prism software (GraphPad Software Inc., La Jolla, CA). Data are presented as means ± SEM and statistical significance was assessed using unpaired T-tests (GraphPad Prism).

### Chemokine Competition Binding

MIP-1β was radio-iodinated using the chloramine T method as previously described [36], [37]. HEK 293 cells (3×10^6/^10 cm dish), transiently transfected with wild type or mutant CCR5 receptor constructs were detached (5 mM EDTA, 50 mM HEPES, pH 7.4, 100 mM NaCl), re-suspended (3×10^5^ cells/tube) in binding buffer (50 mM HEPES, pH 7.4, 1 mM CaCl_2_, 5 mM MgCl_2_, 0.5% BSA) and incubated, in triplicate, with [^125^I]-MIP-1β (50 000 cpm, approximately 0.05 pmol) and increasing concentrations of unlabelled MIP-1β (0 to 10^−7 ^M) in a total volume of 0.2 ml (60 min, 27°C), as previously described [22], [37]. Bound tracer was separated by filtration through glass-fiber filters (GF/C, Whatman, Maidstone, England) presoaked in 1% BSA. Filters were washed twice with washing buffer (50 mM HEPES, pH 7.4, 1 mM CaCl_2_, 5 mM MgCl_2_ and 0.5 M NaCl) and radioactivity was counted in a γ-counter. Total binding (B_0_) of [^125^I]-MIP-1β to the receptor was determined in the absence of unlabeled ligand, whereas non-specific binding (NSB) was determined as the amount of radio-labeled ligand bound in the presence of 10^−7 ^M unlabeled MIP-1β or bound to untransfected cells. Specific binding of [^125^I]-MIP-1β was calculated as the difference between B_0_ and NSB. Concentrations of MIP-1β that displaced 50% of total specific [^125^I]-MIP-1β binding (IC_50_ values) were calculated using GraphPad Prism and nonlinear regression for one-site competition curves. Data are presented as means ± SEM and statistical analysis of pIC_50_ values was performed using unpaired two-tailed T-tests.

### Fluorescence-Activated Cell Sorting (FACS) Analysis of CCR5 Receptor Expression

HEK 293 or HOS-CD4-Luc cells transfected with wild type or mutant CCR5 constructs were detached from the 10 cm^2^ dishes, suspended in 10 ml of phosphate-buffered saline containing BSA (PBS-BSA, 137 mM NaCl, 2.7 mM KCl, 1.4 mM KH_2_PO_4_ and 4.3 mM Na_2_HPO_4_.7H_2_O, pH 7.3, 0.5% BSA) and centrifuged (1000 rpm, 10 min). The cell pellet was re-suspended in PBS-BSA (0.5 ml) and re-suspended cells (20 µl) were incubated with phycoerythrin-labeled 2D7 mouse anti-hCCR5 antibody (PE-2D7, BD BioSciences Pharmingen, Franklin Lakes, NJ, 50 ng, 21°C, 60 min) in the dark. Samples were centrifuged (2000 rpm, 10 min), washed in PBS-BSA (1.5 ml) and re-suspended in PBS-BSA (500 µl) for FACS analysis using a FACScalibur flow cytometer (Becton-Dickinson, Franklin Lakes, NJ). Untransfected HEK 293 cells stained with PE-2D7 were used as a negative control to set the gating threshold and the mean fluorescence of gated cells transfected with the wild type construct was defined as 100% for each experiment.

### Env-Directed Cell Fusion Assay

A cell fusion assay that models the interaction of the host cell receptors with the Env protein expressed on the membrane of the HIV-1 virion [32] was used to assess the ability of mutant receptors to mediate Env-dependent membrane fusion. In this assay, HEK 293 cells expressing HIV Env protein and the HIV transcription factor, Tat, were mixed with HOS-CD4-Luc reporter cells expressing CCR5 receptors. Binding of Env on the HEK 293 cells to CD4 and CCR5 on the transfected HOS-CD4-Luc cells allows fusion of the cells and Tat expressed in HEK 293 cells is able to activate Luc expression via the LTR promoter in the HOS-CD4-Luc cells.

HOS-CD4-Luc cells were transiently transfected with wild type or mutant CCR5 receptor cDNA cloned into the hygromycin resistant vector, pcDNA3.1/Hygro(+) (Invitrogen), cultured overnight and then cultured (48 h) in DMEM supplemented with FCS (10%), G418 (400 µg/ml) and hygromycin (200 µg/ml, Sigma, St. Louis, Missouri) to select for transfected cells. Expression of CCR5 was assessed by FACS analysis and HOS-CD4-Luc cells expressing wild type or mutant CCR5 constructs were seeded into 96-well plates (Corning, 6 000 cells/well). HEK 293 cells transfected with Du151 gp150 *env* [31], *rev* and *tat* 24 h after transfection of HOS-CD4-Luc cells were layered at increasing densities (30 cells/well –48 000 cells/well in triplicate) onto transfected HOS-CD4-Luc cells and co-cultured overnight to allow cell fusion. Luciferase activity was determined using the luciferase assay system (Promega, Madison, WI) according to the manufacturer’s instructions and a Veritas luminometer (Promega).

## Results

### Effects of Amino Acid Substitutions on CCR5 Receptor Signaling

**Figure 1:**
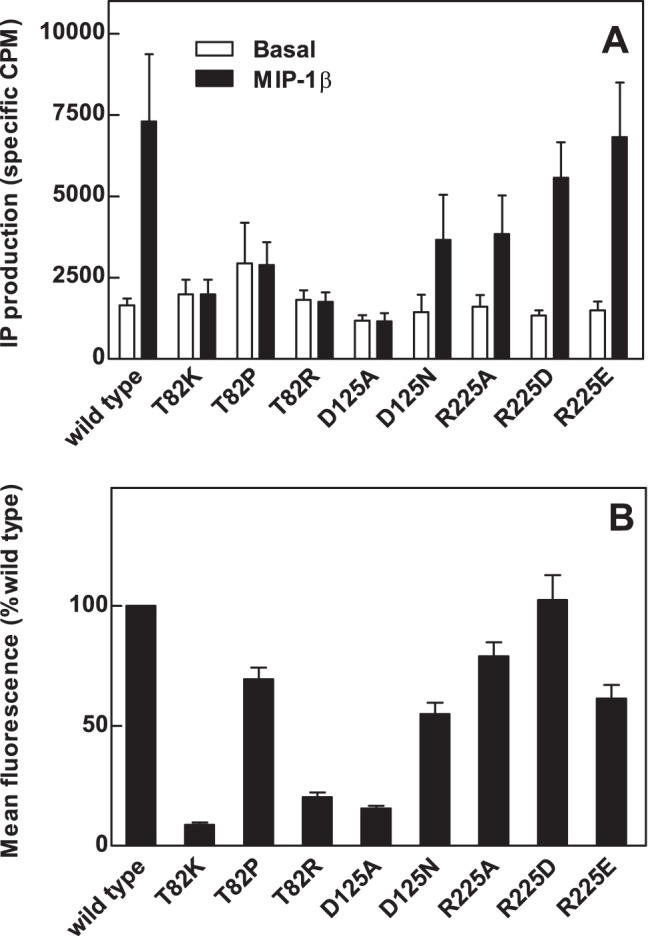
IP production and expression of wild type and mutant CCR5 receptors. HEK-Gqi cells were transiently transfected with wild type or mutant CCR5 receptors, labeled with [^3^H]*myo*-inositol and incubated without (basal) or with chemokine agonist, MIP-1β (10^−7^ M). Specific CPM denotes the CPM determined for receptor expressing-cells minus the CPM for vector-transfected cells. Data are from a representative experiment performed at least three times in duplicate. B, HEK 293 cells transiently transfected with wild type or mutant CCR5 receptors were stained with a PE-2D7 anti-CCR5 antibody and analyzed by FACS. Data are representative of at least three independent experiments performed in duplicate.

**Figure 2:**
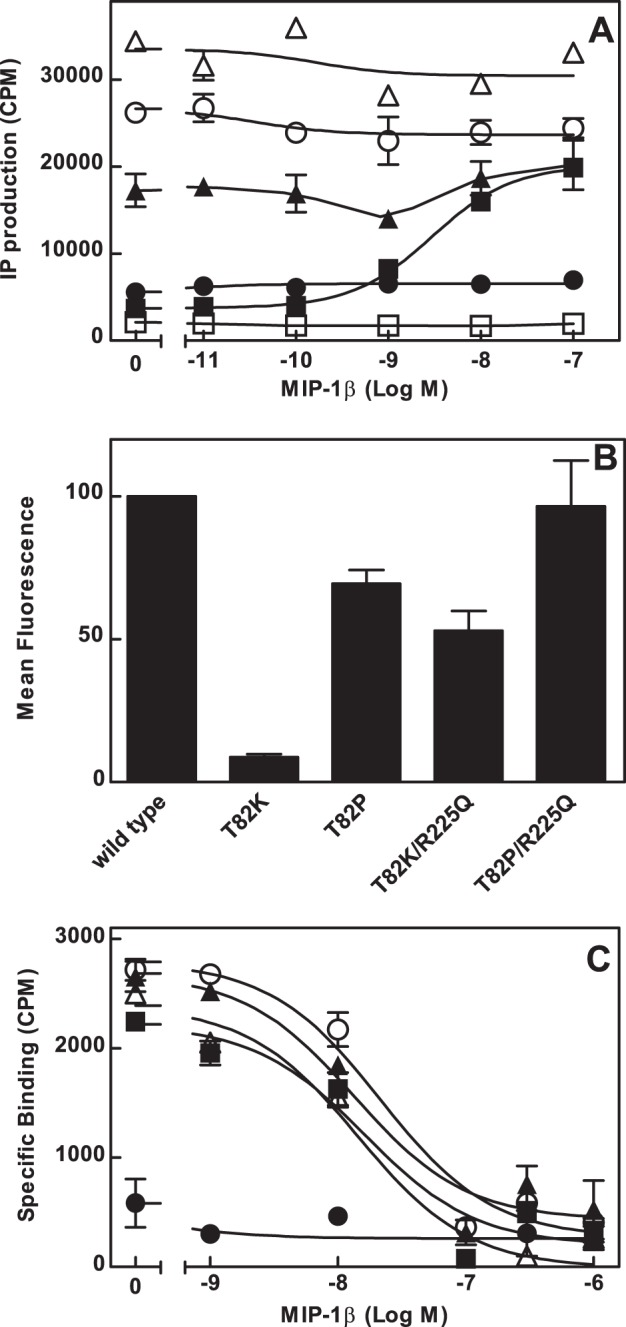
IP production, expression and competition binding of CCR5 receptors with mutations of Thr^2.56(82)^ and Arg^6.32(225)^. A, HEK-Gqi cells were transfected with the wild type (▪) or mutant CCR5 receptors Thr^2.56(82)^Lys (•), Thr^2.56(82)^Pro (▴), Thr^2.56(82)^Lys/Arg^6.32(225)^Gln (○) or Thr^2.56(82)^Pro/Arg^6.32(225)^Gln (Δ). Untransfected cells (□) were used as a negative control. Cells pre-labeled with [^3^H]*myo*-inositol were incubated with increasing concentrations of MIP-1β. Data are from a single experiment that is representative of at least three independent experiments performed in duplicate. B, HEK cells were transfected with wild type or mutant CCR5 receptors and stained with PE-2D7 for FACS analysis. Results are mean values ± SEM from at least three independent experiments performed in duplicate. C, HEK 293 cells were transiently transfected with wild type (▪) or mutant CCR5 receptors, Thr^2.56(82)^Lys (•), Thr^2.56(82)^Pro (▴), Thr^2.56(82)^Lys/Arg^6.32(225)^Gln (○) or Thr^2.56(82)^Pro/Arg^6.32(225)^Gln (Δ) and incubated with ^125^I-MIP-1β and various concentrations of unlabelled MIP-1β. Cell-bound radioactivity was collected by filtration and counted. Data are from a single experiment, representative of at least three independent experiments performed in triplicate.

Eight mutant CCR5 receptor constructs that were predicted to be constitutively active were prepared and examined for constitutive and agonist-stimulated IP production in HEK-Gqi cells. Cells expressing the wild type CCR5 receptor displayed increased basal IP production compared to vector-transfected cells (data not shown) and showed enhanced IP production in response to MIP-1β (10^−7^ M, Fig. 1A, Table 1). All mutants with substitutions of the Thr^2.56(82)^ residue displayed enhanced basal IP production compared with the wild type receptor (Fig. 1A, Table 1), consistent with a previous report that these mutants are constitutively active [21]. All three mutants showed no further increase in IP production in response to MIP-1β (Fig. 1A, Table 1). Basal IP production in cells transfected with wild type CCR5 or mutant receptors varied with transfection efficiency (compare Figs. 1A and 2A), which resulted in relatively large SEM values (Table 1). The “DRY” motif mutants, Asp^3.49(125)^Ala and Asp^3.49(125)^Asn, displayed basal IP production that was similar to wild type levels, but displayed decreased IP production in response to MIP-1β (Fig. 1A, Table 1), suggesting that these mutants may be either poorly expressed or uncoupled from G protein activation. The third intracellular loop mutants, Arg^6.32(225)^Ala, Arg^6.32(225)^Asp and Arg^6.32(225)^Glu, displayed basal IP production that was comparable with wild type IP production and decreased MIP-1β-stimulated IP production (Fig. 1A, Table 1), showing that they also were not more constitutively active than wild type CCR5.

**Table 1:** IP production and surface expression of wild type and mutant CCR5 receptors.

	IP Production		FACS analysis	
CCR5 Receptor Construct	Basal	Stimulated	Mean Fluorescence Intensity	Cells gated
	(CPM)	(CPM)	(% wild type)	(%)
**Wild type**	2 263±417 (9)	15 684±1 198	100	86±0.5
**Thr^2.56(82)^Lys**	4 783±1 007^a^ (9)	4 516±915	6±1.5	8±0.5
**Thr^2.56(82)^Pro**	9 004±3284^a^ (6)	12 382±3 161	92±15	47±6.7
**Thr^2.56(82)^Arg**	2 358±373	2 827±802	19±3	51±0.8
**Asp^3.49(125)^Ala**	1 811±368	1 799±680	11±1.7	46±1.8
**Asp^3.49(125)^Asn**	1 338±338	2827±802	47±8.5	74±0.3
**Arg^6.32(225)^Ala**	1 438±360	6 197±2 550	63±10	57±11
**Arg^6.32(225)^Asp**	1 664±259	6 446±1 556	72±24	61±19
**Arg^6.32(225)^Glu**	1 808±418	6 697±2 022	43±12	69±5.0
**T^2.56(82)^K/R^6.32(225)^Q**	14 500±4 321^a^ (4)	14 187±4 320	51±13	48±7.5
**T^2.56(82)^P/R^6.32(225)^Q**	15 540±6 929^a^ (4)	18 038±6 700	80±21	58±7.9

a significantly different from wild type, p<0.05.

To assess constitutive- and ligand-stimulated IP production, HEK-Gqi cells transiently expressing wild type or mutant CCR5 receptors were labeled with [H^3^]-*myo*-inositol and incubated with buffer (Basal) or MIP-1β (10^−7^ M, Stimulated). To assess cell surface expression of receptors HEK 293 cells transiently transfected with wild type or mutant CCR5 constructs were incubated with PE-2D7 antibody before FACS analysis. Every experiment included wild type CCR5 and mock transfected cells. Data are means ± SEM calculated from at least three independent experiments performed in duplicate.

### Effects of Amino Acid Substitutions on CCR5 Receptor Expression

FACS analysis of cell surface CCR5 expression was used to distinguish changes in receptor expression levels and increased constitutive activity as potential causes of altered IP production in cells transfected with mutant CCR5 constructs. Mean fluorescence was used as a measure of the relative density of receptors expressed on individual cells, while the percentage of cells gated indicates the number of cells expressing more than the threshold level of receptor protein. HEK 293 cells were transiently transfected with CCR5 receptor constructs and the mean fluorescence of gated wild type-transfected cells was defined as 100% for each experiment. 86% of cells transfected with the wild type were gated (Table 1), indicating high transfection efficiency for HEK 293 cells. The Thr^2.56(82)^Pro mutant, which showed the highest basal IP production, exhibited mean fluorescence comparable with that of the wild type receptor (Fig. 1B, Table 1). In contrast, the Thr^2.56(82)^Lys mutant receptor, which also showed increased basal IP production, was poorly expressed, exhibiting low mean fluorescence (6±1.5% of wild type levels, Fig. 1B, Table 1) and a low proportion of cells gated (8±0.5%, Table 1). This low expression combined with a high level of ligand-independent IP production suggests that the Thr^2.56(82)^Lys mutant receptor is highly constitutively active. The Thr^2.56(82)^Arg mutant receptor showed intermediate expression levels (Fig. 1B, Table 1). Mutation of Asp^3.49(125)^ to Ala decreased receptor expression, whereas mutation of Asp^3.49(125)^ to Asn or mutation of Arg^6.32(225)^ (Arg^6.32(225)^Asp, Arg^6.32(225)^Ala or Arg^6.32(225)^Glu) had less marked effects on expression of receptor protein (Fig. 1B, Table 1).

### Double Amino Acid Substitutions Enhance Expression of Constitutively Active CCR5 Mutants in HEK 293 Cells

As it is well established that efficiency of Env-dependent HIV fusion with host cells is affected by the number of co-receptors expressed on the cell surface [38], [39], [40], [41], [42], decreased expression of constitutively active CCR5 receptors is a potential confounding factor in using these mutant receptors to assess the role of receptor conformation in Env-directed membrane fusion. Thus, it was necessary to enhance expression of mutant receptors to wild type levels. We initially tried to use the inverse agonist, TAK 779, as a molecular chaperone to increase expression of mutant receptors, but its effects were inconsistent and residual drug was a concern for subsequent analyses. We were also unable to stably express CCR5 constructs in the HOS-CD4-Luc cells. An alternative approach was to combine the mutations that resulted in constitutive activity with the Arg^6.32(225)^Gln mutation, which previously yielded partial constitutive activity without decreasing receptor expression [22]. Cells transfected with the Thr^2.56(82)^Lys/Arg^6.32(225)^Gln double-mutant receptor produced basal IP levels 7.7-fold higher than the wild type receptor and the Thr^2.56(82)^Pro/Arg^6.32(225)^Gln mutant receptor displayed basal IP production 9.3-fold higher than that of the wild type receptor (Fig. 2A, Table 1). MIP-1β did not further increase IP production in cells expressing either mutant (Fig. 2A, Table 1). Basal IP production stimulated by the double mutant receptors was higher than the basal IP production of the single mutants and comparable to the maximum MIP-1β-stimulated IP production of the wild type receptor. FACS analysis confirmed that expression of the Thr^2.56(82)^Lys/Arg^6.32(225)^Gln double-mutant receptor was increased compared with the Thr^2.56(82)^Lys receptor (Fig. 2B, Table 1).

Constitutively active GPCRs often have enhanced affinity for agonist ligands [43]. Homologous competition-binding assays were used to assess the affinity of wild type and mutant receptors for the chemokine MIP-1β. In cells expressing the wild type CCR5 receptor, unlabelled MIP-1β displaced the ^125^I-MIP-1β with an IC_50_ value of 32.6 nM ±6.5 nM (Fig. 2C). The Thr^2.56(82)^Lys receptor showed specific binding that was too low for calculation of an IC_50_ value, consistent with poor expression of this mutant. In contrast, the double mutant, Thr^2.56(82)^Lys/Arg^6.32(225)^Gln, displayed total binding comparable to the wild type receptor with an IC_50_ value of 20.67±4 nM (Fig. 2C). Similarly, both mutants with Pro in position 82, Thr^2.56(82)^Pro and Thr^2.56(82)^Pro/Arg^6.32(225)^Gln, displayed total binding and affinity comparable to the wild type receptor with IC_50_ values of 31.9±7.4 nM and 30.6±13 nM respectively (Fig. 2C). IC_50_ values for the mutant receptors were not significantly different from the wild type receptor.

### Env-Directed Cell Fusion

**Figure 3:**
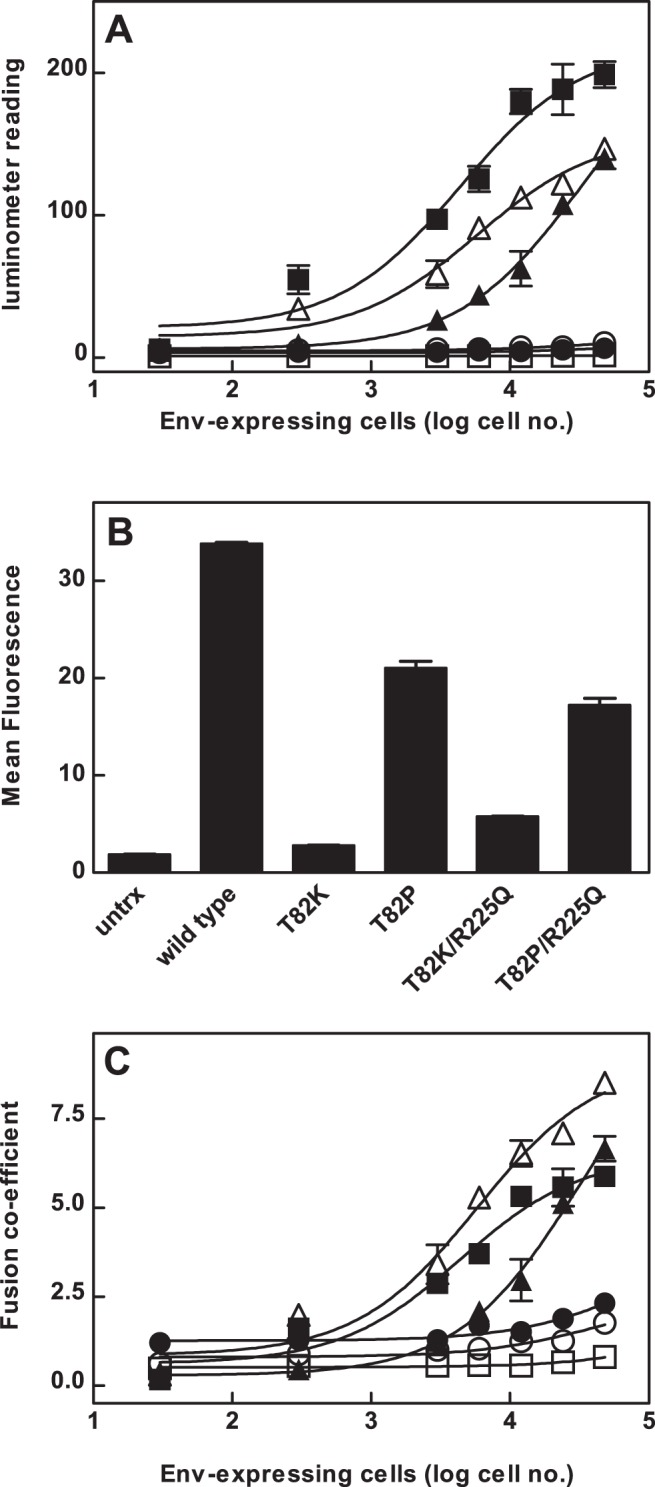
Fusion activity of wild type and mutant CCR5 receptors. A, HOS cells stably expressing CD4 and the luciferase reporter gene were transiently transfected with wild type (▪) or mutant CCR5 receptors Thr^2.56(82)^Lys (•), Thr^2.56(82)^Pro (▴), Thr^2.56(82)^Lys/Arg^6.32(225)^Gln (○) or Thr^2.56(82)^Pro/Arg^6.32(225)^Gln (Δ). CCR5-expressing HOS-CD4-Luc cells were co-cultured overnight with HEK cells transiently expressing tat, rev and Env and luciferase activity was assessed. B, CCR5-expressing HOS-CD4-Luc cells were labeled with PE-2D7 and analyzed by FACS analysis. C, To compare fusion efficiency among mutant receptors that were expressed at different levels the fusion coefficient was derived by dividing the luciferase activity by the mean fluorescence of each construct.

To assess the ability of the constitutively active CCR5 mutant receptors to mediate fusion with cells expressing HIV Env protein, cell fusion assays were performed, using dose-response curves in which Env concentration was varied by varying the numbers of Env-expressing HEK 293 cells, while the concentrations of receptor-expressing HOS-CD4-Luc cells were held constant. This is analogous to standard dose-response experiments with the Env-expressing cells constituting the agonist ligand. Cells expressing the wild type CCR5 receptor fused well with Env-expressing cells (Fig. 3A) and exhibited a mean EC_50_ value of 14,705±4591 Env-expressing cells/well (Table 2). Mutant receptors with Lys in position 82, Thr^2.56(82)^Lys and Thr^2.56(82)^Lys/Arg^6.32(225)^Gln, both mediated very low levels of Env-directed fusion (Fig. 3A, Table 2). In contrast, cells expressing mutants with Pro in position 82, Thr^2.56(82)^Pro and Thr^2.56(82)^Pro/Arg^6.32(225)^Gln, displayed high levels of Env-directed fusion that were comparable with that mediated by the wild type receptor (Fig. 3A, Table 2). The EC_50_ value for the Thr^2.56(82)^Pro mutant was similar to wild type (Table 2) and the EC_50_ value for the Thr^2.56(82)^Pro/Arg^6.32(225)^Gln double mutant was lower (Table 2).

**Table 2:** Env-directed membrane fusion mediated by wild type and mutant CCR5 receptors.

CCR5 Receptor Construct	Maximum Fusion	Maximum Fusion Efficiency	EC_50_
	(% wild type)	(fusion coefficient)	(Env-expressing cells/well)
**Wild Type**	100	11.8±2.2	14,705±4,591
**T^2.69(82)^K**	4.6±1.7	0.84±0.2	ND^a^
**T^2.69(82)^P**	135±10.9	16.5±4.1	15,384±4,818
**T^2.69(82)^K/R^6.32(225)^Q**	16.8±3.1	1.9±0.4	ND^a^
**T^2.69(82)^P/R^6.32(225)^Q**	134.8±31.1	18.8±5.6	5,545±957

a ND, not determined because maximum fusion was too low to allow determination of EC_50_.

HOS-CD4-Luc cells expressing wild type or mutant CCR5 receptor constructs were co-cultured with increasing concentrations of HEK 293 cells expressing HIV Env and the HIV transactivator, tat, and luciferase activity was measured. Data are means ± SEM of at least five experiments performed in triplicate.

FACS analysis showed that mutant CCR5 receptors were expressed at levels lower than wild type CCR5 in HOS-CD4-Luc cells (Fig. 3B). As we were unable to generate HOS-CD4-Luc cell lines stably expressing mutant CCR5 receptors, we calculated a fusion efficiency coefficient to take account of differences in receptor expression (Fig. 3C, Table 2). The wild type CCR5 receptor showed a maximum fusion coefficient of 11.8±2.2. The Pro-containing mutants, Thr^2.56(82)^Pro and Thr^2.56(82)^Pro/Arg^6.32(225)^Gln, showed high maximum fusion coefficients of 16.5±4.1 and 18.8±5.6 respectively (Fig. 3C, Table 2). In contrast, the Lys-containing mutants, Thr^2.56(82)^Lys and Thr^2.56(82)^Lys/Arg^6.32(225)^Gln, both showed very low maximum fusion coefficients (Fig. 3C, Table 2). These results show that CCR5 mutants that constitutively activate IP signaling fall into two categories, those with Lys in position 82 are poor mediators of fusion, whereas those with Pro in position 82 are good mediators of fusion. The two classes of constitutively active mutants may define distinct activated-receptor conformations that differ in their interactions with HIV Env protein.

In summary, we generated four CCR5 mutants that constitutively activate IP signaling. The Thr^2.56(82)^Pro and Thr^2.56(82)^Pro/Arg^6.32(225)^Gln mutants, which were expressed at levels similar to the wild type receptor in HEK 293 cells, the Thr^2.56(82)^Lys mutant, which was poorly expressed, and the double mutant, Thr^2.56(82)^Lys/Arg^6.32(225)^Gln, which showed enhanced expression relative to the Thr^2.56(82)^Lys mutant. Constitutively active mutants with Lys in position 82 showed very low fusion efficiency, but mutants with Pro in position 82 showed good fusion efficiency that was comparable to the wild type receptor.

## Discussion

We have investigated the ability of activated CCR5 conformations to mediate HIV Env-directed membrane fusion by generating constitutively active mutant CCR5 receptors. Charge-neutralizing substitutions for Asp^3.49(125)^ in the DRY motif and substitutions of the naturally occurring Arg^6.32(225)^Gln mutation of CCR5 did not increase constitutive activation of IP signaling. However, substitution of the Thr^2.56(82)^ residue of the TxP motif caused high levels of ligand-independent cellular signaling. The Thr^2.56(82)^Lys mutation also decreased cell surface CCR5 protein. Severely decreased expression of mutants with Lys, but not Pro, in position 82 suggests that the conformations of the constitutively active mutant receptors differ. Mutant CCR5 receptors with Lys in position 82, which constitutively activated IP signaling, were poor mediators of Env-directed membrane fusion, suggesting that HIV might not enter cells via the activated receptor conformation. However, constitutively active receptors with Pro substituted into the TxP motif mediated Env-directed membrane fusion very efficiently. The differential effects on receptor expression and membrane fusion suggest that Lys and Pro substitutions in position 82 stabilize distinct activated conformations of CCR5 that vary in their ability to mediate Env-dependent membrane fusion.

Constitutively active GPCR mutants are defined by increased ligand-independent (basal) signaling activity. The increased signaling results from an increased population of activated receptor conformations by mutant receptors. Many constitutively active mutants exhibit decreased cell surface expression, which may result from constitutive internalization of the activated receptor, or from increased flexibility of activated receptor conformations that results in protein instability [44], [45], [46], [47].

Inactive and agonist-stabilized activated conformations of CCR5 are likely to be broadly similar to those of other family A GPCRs for which crystal structures are known, including the closely related CXCR4 chemokine receptor [10], [48]. Highly conserved amino acid motifs are likely to form intramolecular interactions in CCR5 that are similar to the interactions formed in the inactive and active conformations of GPCRs that have been crystallized [17], [49], [50].

The “DRY” motif, at the cytosolic end of TMS3, is one of the most conserved sequences among class A GPCRs. According to the “consensus” view of its function, the basic side-chain of Arg^3.50^ interacts simultaneously with the adjacent acidic Asp^3.49^ (Glu^3.49^ in rhodopsin) and with Glu^6.30^ at the cytosolic end of TMS6, forming an “ionic lock” that stabilizes inactive receptor conformations. In activated receptors the ionic lock is broken and the guanidino group of Arg^3.50^ moves to interact with Tyr^5.58^ in TMS5 [17], [49], [50], [51]. The switch function of the DRY motif is supported by charge-neutralizing mutations of Asp^3.49^ or Arg^3.50^, which cause constitutive activity in many GPCRs [19], [51]. However, our charge-neutralizing mutations of Asp^3.49(125)^ did not cause constitutive activation of IP signaling in CCR5. Decreased expression of the mutant receptors suggests that substitution of Asp^3.49(125)^ decreases receptor protein stability or increases receptor internalization and down-regulation, as has been described for the Arg^3.50(126)^Asn CCR5 mutant [52]. This suggests that the role of the DRY motif in activation of CCR5 does not comply with the consensus view [51], [53].

The Glu^6.30^ residue in intracellular loop 3 forms part of the ionic lock in rhodopsin, but many GPCRs, including CCR5, have basic residues in position 6.30 [53]. Crystal structures of the inactive CXCR4 chemokine receptor show no interaction between Arg^3.50^ and Arg^6.30^ [10], [50]. The naturally-occurring Arg^6.32(225)^Gln CCR5 mutant is partially constitutively active and we hypothesized that Arg^6.32(225)^, which is two residues away from Arg^6.30(223)^, might form alternative interactions that stabilize the inactive CCR5 conformation. Other mutations of Arg^6.32(225)^ did not increase constitutive activity. Decreased expression of these mutants is consistent with the role of basic amino acids in stabilizing membrane-spanning helices [54] although the naturally-occurring Arg^6.32(225)^Gln mutation did not decrease receptor expression [22]. Furthermore, combining the Thr^2.56(82)^Lys and Thr^2.56(82)^Pro mutations with the Arg^6.32(225)^Gln mutation increased expression of constitutively active mutant CCR5 receptors. The Arg^6.32(225)^Gln mutation may stabilize a receptor conformation that is less susceptible to internalization or to degradation. The Arg^6.32(225)^Gln double mutation enhanced expression of constitutively active receptors more effectively in HEK 293 cells than in HOS-CD4-Luc cells. This may result from different receptor trafficking in the two cell lines or it may reflect the generally lower transfection efficiency and receptor expression in HOS-CD4-Luc cells.

A proposal that the TxP motif acts as a switch that activates CCR5 was supported by mutations that uncoupled the CCR5 receptor from cellular signaling [20], [55] or increased constitutive cellular signaling [21]. The Thr^2.56(82)^Lys and Thr^2.56(82)^Pro CCR5 mutants that we tested displayed increased basal IP production and could not be further stimulated by MIP-1β. The same mutants were constitutively active and showed no further response to chemokine treatment in a yeast reporter system [21], suggesting that they are fully stabilized in activated conformations. They also constitutively stimulated GTPγS binding in stably transfected CHO cells. However, agonist treatment enhanced GTPγS binding [21], suggesting that the Thr^2.56(82)^Lys and Thr^2.56(82)^Pro mutations do not fully stabilize the CCR5 conformation that activates the cognate Gαi protein. The double mutants, Thr^2.56(82)^Lys/Arg^6.32(225)^Gln and Thr^2.56(82)^Pro/Arg^6.32(225)^Gln, both showed basal IP production that was similar to the maximum MIP-1β-stimulated IP production mediated by wild type CCR5, suggesting that they are fully stabilized in activated conformations. However, it is not known whether the CCR5 conformations that activate native Gαi signaling pathways are fully stabilized in the double mutant receptors. Mutant receptors with Lys substituted for Thr^2.56(82)^ showed decreased cell surface protein, which may result from decreased receptor stability or stabilization of receptor conformations that constitutively expose cytosolic Ser residues to G protein-coupled receptor kinases, leading to constitutive internalization [44], [45], [46], [47], [56]. In contrast, the Thr^2.56(82)^Pro mutation may stabilize receptor conformations that are not recognized by receptor kinases or are less flexible. The differential expression suggests that constitutively active CCR5 mutants with Pro or Lys in position 2.56(82) may be stabilized in distinct conformations that are differentially sensitive to internalization and/or degradation. Distinct receptor conformations of the Thr^2.56(82)^Lys and Thr^2.56(82)^Pro CCR5 mutants is supported by the report that CHO cells expressing the Thr^2.56(82)^Pro CCR5 mutant exhibited a wild type-like chemotactic response to the chemokine ligand, RANTES, whereas cells expressing the Thr^2.56(82)^Lys mutant showed no chemotactic response [21].

The extended ternary complex model of receptor activation predicts that constitutively active receptors have increased agonist binding affinity, even in the absence of G protein [43]. However, some constitutively active receptors do not exhibit this phenotype [57], [58]. We did not find significant changes in IC_50_ values for MIP-1β binding to constitutively active CCR5 mutants. Arias *et al* reported similar results for MIP-1β binding, but found that the Thr^2.56(82)^Lys mutation decreased affinity for the agonist chemokines, MIP-1α and RANTES, whereas the Thr^2.56(82)^Pro mutation had less effect [21]. Studies with small molecule drugs have suggested that the different chemokine ligands interact with distinct CCR5 conformations [59], [60]. The Thr^2.56(82)^Lys mutation may selectively destabilize the ensembles of CCR5 conformations that preferentially bind MIP-1α and RANTES.

The gp120 subunit of HIV Env is a CCR5 receptor agonist [6], [7], [8]. However, Env mediates membrane fusion in cells expressing mutant CCR5 receptors that do not support chemokine-stimulated signaling [23], [24], [25], suggesting that inactive conformations of CCR5 mediate membrane fusion. Furthermore, HIV isolates that are resistant to CCR5 blockers use drug-occupied CCR5 that is stabilized, by the inverse agonist drug, in the inactive conformation to infect cells. We therefore hypothesized that an inactive CCR5 conformation mediates HIV infection and that activated conformations of CCR5 may not support HIV Env-directed membrane fusion.

Consistent with our hypothesis, both of the constitutively active mutants with Lys in position 82 showed low Env-directed membrane fusion efficiency. The decreased fusion may result from decreased expression, as the Thr^2.56(82)^Lys/Arg^6.32(225)^Gln double mutation did not fully recover expression in the HOS-CD4-Luc cells used for the fusion assay. Fusion remained lower than that mediated by wild type CCR5 after correction for receptor expression, but we cannot exclude threshold effects of receptor protein levels. In contrast, constitutively active CCR5 receptors with Pro in position 82 mediated membrane fusion similar to that mediated by wild type CCR5. Our results suggest that CCR5 receptors that constitutively activate IP signaling exist in at least two distinct conformations. One conformation, stabilized by Pro in position 82, supports Env-directed membrane fusion, whereas the other conformation, stabilized by Lys in position 82, does not.

**Figure 4:**
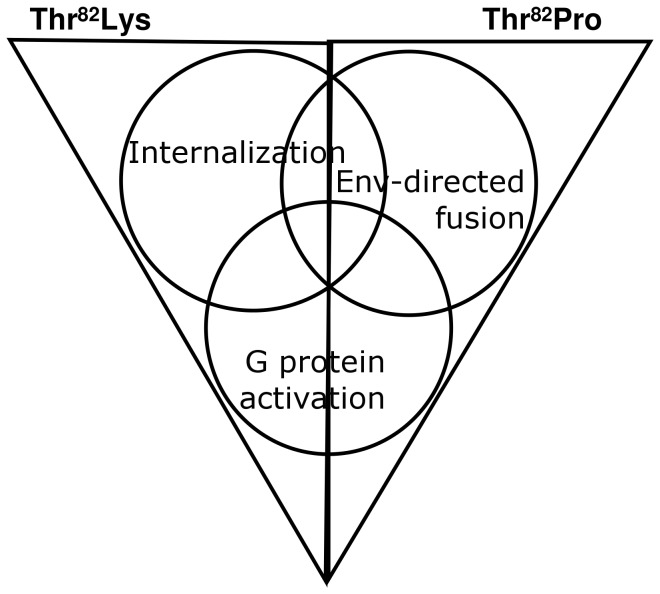
Venn diagram depicting ensembles of CCR5 receptor conformations stabilized by mutation of Thr^2.56(82)^. Triangles represent receptor conformations stabilized by mutation of Thr^2.56(82)^ to Lys or Pro. Circles represent receptor conformations that mediate G protein activation, receptor internalization or HIV Env-directed membrane fusion. Mutation of Thr^2.56(82)^ to Lys stabilizes an ensemble of receptor conformations that activate G protein-mediated signaling and conformations with increased susceptibility to internalization, but not conformations that support HIV Env dependent membrane fusion. The Thr^2.56(82)^Pro mutation stabilizes an ensemble of receptor conformations that activate the G protein and conformations that support HIV-1 fusion, but it does not appear to increase population of receptor conformations that result in decreased membrane expression of CCR5.

The different capacities of constitutively active CCR5 receptors to mediate membrane fusion may relate to the nature of their constitutive activity. Decreased expression of mutants with Lys in position 82 suggests constitutive receptor phosphorylation and activation of receptor sequestration pathways [61]. Constitutive internalization of CCR5 may target CCR5-Env complexes for degradation and thus inhibit the membrane fusion pathway. Alternatively, receptor conformations that are stabilized by Lys in position 82 may have decreased affinity for HIV Env or decreased ability to induce the fusogenic Env protein conformation that mediates membrane fusion. In terms of the ensemble model of receptor conformation [3], [62], mutation of Thr^2.56(82)^ to Lys, may stabilize an ensemble of CCR5 conformations that includes the micro-conformations that activate G proteins and receptor internalization, but not the micro-conformations that induce Env-directed membrane fusion. In contrast, mutation of Thr^2.56(82)^ to Pro appears to stabilize an ensemble of receptor conformations that activate G protein and mediate the co-receptor functions of CCR5, but do not activate internalization (Fig. 4).

Distinct activated conformations of CCR5 with differential abilities to support HIV Env-directed membrane fusion opens the possibility of developing CCR5 ligands that select specific receptor conformations. Indeed, a recent comparison of the CCR5 blockers, TAK 779 and maraviroc, has shown that maraviroc has higher antiviral potency that does not correlate with inverse agonist activity or ability to block gp120 binding. It was suggested that maraviroc may selectively destabilize CCR5 conformations that trigger Env penetration of cell membranes [63]. Furthermore, it has been shown that CCR5 heterodimerizes with the CXCR4 co-receptor and that antagonists specific for one receptor allosterically cross-inhibit ligand binding and agonist function at the other receptor [64]. This raises the potential that CCR5-blocking drugs may be developed to cross-inhibit infection by X4-tropic viruses in cells where both receptors are expressed.

In conclusion, we have shown that charge-neutralizing mutations of the Asp^3.49(125)^ residue of the DRY motif do not result in constitutive activity of CCR5, confirming that the CCR5 receptor does not conform to the consensus mechanism of receptor activation. We have confirmed that Lys or Pro substitutions for the Thr^2.56(82)^ residue of the TxP motif cause constitutive activity of CCR5, but we have shown that mutants have distinct properties. Constitutively active mutants with Lys in position 82 show decreased cell surface expression and decreased HIV co-receptor function, whereas mutants with Pro in position 82 were well expressed and fully functional as HIV co-receptors. These distinct properties suggest that the mutations stabilize ensembles of receptor conformations that differ in their ability to induce the fusogenic HIV Env conformation. Our results suggest that drugs that stimulate internalization of CCR5 may effectively inhibit HIV infection, both by decreasing cell surface expression of CCR5 and by stabilizing receptor conformations that inhibit fusion of virus that binds to drug-occupied receptor.
